# Predictors of Fatigue Severity in Early Systemic Sclerosis: A Prospective Longitudinal Study of the GENISOS Cohort

**DOI:** 10.1371/journal.pone.0026061

**Published:** 2011-10-14

**Authors:** Shervin Assassi, Astrud L. Leyva, Maureen D. Mayes, Roozbeh Sharif, Deepthi K. Nair, Michael Fischbach, Ngan Nguyen, John D. Reveille, Emilio B. Gonzalez, Terry A. McNearney, for the GENISOS Study Group

**Affiliations:** 1 Department of Medicine, Division of Rheumatology, University of Texas Health Science Center at Houston, Houston, Texas, United States of America; 2 Department of Medicine, University of Texas Medical Branch at Galveston, Galveston, Texas, United States of America; 3 Department of Medicine, Division of Rheumatology, University of Texas Health Science Center at San Antonio, San Antonio, Texas, United States of America; University of Pittsburgh, United States of America

## Abstract

**Objectives:**

Longitudinal studies examining the baseline predictors of fatigue in SSc have not been reported. Our objectives were to examine the course of fatigue severity over time and to identify baseline clinical, demographic, and psychosocial predictors of sequentially obtained fatigue scores in early SSc. We also examined baseline predictors of change in fatigue severity over time.

**Methods:**

We analyzed 1090 longitudinal Fatigue Severity Scale (FSS) scores belonging to 256 patients who were enrolled in the Genetics versus Environment in Scleroderma Outcomes Study (GENISOS). Predictive significance of baseline variables for sequentially obtained FSS scores was examined with generalized linear mixed models. Predictors of change in FSS over time were examined by adding an interaction term between the baseline variable and time-in-study to the model.

**Results:**

The patients' mean age was 48.6 years, 47% were Caucasians, and 59% had diffuse cutaneous involvement. The mean disease duration at enrollment was 2.5 years. The FSS was obtained at enrollment and follow-up visits (mean follow-up time = 3.8 years). Average baseline FSS score was 4.7(±0.96). The FSS was relatively stable and did not show a consistent trend for change over time (p = 0.221). In a multivariable model of objective clinical variables, higher Medsger Gastrointestinal (p = 0.006) and Joint (p = 0.024) Severity Indices, and anti-U1-RNP antibodies (p = 0.024) were independent predictors of higher FSS. In the final model, ineffective coping skills captured by higher Illness Behavior Questionnaire scores (*p*<0.001), higher self-reported pain (p = 0.006), and higher Medsger Gastrointestinal Severity Index (p = 0.009) at enrollment were independent predictors of higher longitudinal FSS scores. Baseline DLco % predicted was the only independent variable that significantly predicted a change in FSS scores over time (p = 0.013), with lower DLco levels predicting an increase in FSS over time.

**Conclusions:**

This study identified potentially modifiable clinical and psychological factors that predict longitudinal fatigue severity in early SSc.

## Introduction

Systemic sclerosis (scleroderma, SSc) is an autoimmune disease in which fibrosis of the skin and internal organs occurs in association with small vessel vasculopathy and autoantibody production. Organ-specific and non-organ specific impairments lead to a spectrum of mild to severe limitations in physical, work and social activities, ultimately influencing health-related quality of life [1]–[3]. Fatigue is increasingly recognized as a common debilitating symptom reported by patients with SSc [4]–[7]. Fatigue was rated by SSc patients as the most bothersome symptom [8]. In a Canadian National survey, SSc patients considered fatigue as their most prevalent symptom that had at least moderate impact on activities of daily living [9]. The fatigue severity among SSc patients is similar to fatigue experienced by patients with rheumatoid arthritis (RA), ankylosing Spondylitis, and systemic lupus erythematosus (SLE) [10].

In a large cross sectional study, gastrointestinal (GI) symptoms, perceived dyspnea, number of comorbidities and current smoking were significant correlates of four fatigue related items collected as part of the vitality domain of the SF-36 [4] . To our knowledge, there are no published longitudinal studies of fatigue severity and its predictors in SSc.

The pathophysiology of fatigue in chronic diseases is not well understood, although several causative factors have been identified. These include anemia, malnutrition, nausea and other GI symptoms, cytokine imbalance, sleep disturbances, deconditioning, lifestyle and psychological factors [11].

In the current study, characteristics of fatigue were prospectively measured in SSc patients enrolled in the Genetics versus ENvironment In Scleroderma Outcomes Study (GENISOS) cohort, using the 29-item Fatigue Severity Scale (FSS) [12]. The FSS was designed for determining the impact of fatigue symptoms and severity in chronic diseases and has been extensively utilized in SLE and multiple sclerosis [12]–[14].

The objectives of current study were to examine the course of fatigue severity over time and to identify the baseline demographic, clinical, and psychosocial factors that predict sequentially obtained fatigue scores in early SSc. Furthermore, we examined the predictive significance of the baseline variables for the rate of change in FSS over time.

## Methods

GENISOS is a multicenter prospective study of patients with early SSc. It is conducted at three sites: the University of Texas Medical Branch at Galveston (UTMB), the University of Texas Health Science Center at Houston (UTHSC-H), and the University of Texas Health Science Center at San Antonio (UTHSC-SA). Study recruitment started in January 1998 and is ongoing. The institutional review boards of all participating sites approved the study and written informed consent was obtained according to the declaration of Helsinki from all subjects. The description of the study, cost, risks and discomforts, benefits, and study withdrawal were included in the informed consent. Study investigators and coordinators interviewed all study subjects at each study site.

### Patient Selection

Details of patient recruitment have been formerly reported [15]–[18]. Patients were enrolled if they met the following criteria: 1) age ≥18 years; 2) diagnosis according to the American College of Rheumatology (formerly the American Rheumatism Association) criteria or at least 3 of the 5 CREST syndrome features (**C**alcinosis, **R**aynaud's Phenomenon, **E**sophageal dysmotility, **S**clerodactyly, **T**eleangiectasia) ; 3) disease onset (defined as the time of onset of the first non-Raynaud's symptom) within five years of enrollment; and 4) defined ethnicity. All enrolled patients at the time of analysis were included in this study.

### Data collection

As previously described [15]–[18], the demographic information, clinical manifestations, patient-reported clinical and psychosocial data were obtained at the baseline visit and then on subsequent semi-annual visits.

#### Outcome variable

Fatigue was ascertained with Fatigue Severity Scale (FSS), a 29-item validated questionnaire [12] that reflects how fatigue influences motivation, exercise, physical functioning, daily activities, interference with work, family, or social life. Each item is scored on a scale of zero (completely disagree) to seven (completely agree). A higher score indicates more fatigue severity. The final score is the average of all scores, ranging from 0 to 7. Each patient answered the questionnaire at enrollment and subsequent follow-up visits.

#### Independent variable

To determine the predictors of fatigue severity in the course of disease, we investigated a comprehensive array of potential independent variables from the following domains: demographic information, clinical manifestations, patient-reported clinical and psychosocial data.

#### Demographic information

Age, gender, ethnicity, marital status, educational level, and health habits were recorded. Marital status data were dichotomized as being married or in a marriage-like relationship of cohabitation versus being single, divorced, separated, or never married. We categorized the educational level as holding an associate degree (2 years of college education) and above versus high school diploma and below. Moreover, patients were interviewed by the study coordinators, about their smoking and exercise habits, at each visit. Specifically, patients were asked whether they are currently exercising or smoking cigarettes.

#### Clinical manifestations

Disease type based on the extent of skin involvement [19], duration, and antibody profile were recorded. The disease duration was determined by the study investigators based on patient interview or review of medical records utilizing two different methods: from the first non-Raynaud's phenomenon symptom attributable to SSc and from the first symptom attributable to SSc (Raynaud's or non- Raynaud's phenomenon symptoms) to the time of visit. History, physical examination findings, modified Rodnan Skin Score (mRSS) [20], and Medsger Severity Index (SI) [21] were recorded. Laboratory studies, EKG, and pulmonary function tests (PFT) were obtained at enrollment and annually thereafter. SSc cardiac involvement was defined as having clinically significant arrhythmia (arrhythmia requiring treatment) or ejection fraction ≤40%. All pulmonary function tests were reviewed by a pulmonologist and studies that did not fulfill the American Thoracic Society/European Respiratory Society (ATS/ERS) were excluded [22]. Myositis was diagnosed if the patient had proximal muscle weakness with at least one of the following: elevated levels of muscle enzymes, myopathic changes on electromyography, and/or a characteristic muscle biopsy. Furthermore, we calculated the number of co-morbid conditions in each patient based on the patients' history of cardiovascular disease, hypertension, diabetes mellitus, stroke, lung disease, malignancy, kidney disease, SLE, RA, thyroid disease, osteoarthritis, fibromyalgia, peptic ulcer disease, obesity (body mass index ≥30), depression, and other neuropsychiatric disorders.

#### Patient-reported clinical outcomes

We recorded pain and dyspnea on visual analogue scales (length 10 cm). The anchors of the VAS were 0 (no pain or shortness of breath) to 100 (very severe pain or shortness of breath).The severity of symptoms was measured with a metric ruler in centimeters. A higher score indicated more severe pain or dyspnea.

Patient-reported dyspnea was investigated only in the univariable model. This variable was not included in the subsequent multivariable models because we assumed that there is a strong bi-directional relationship between the perceived dyspnea and fatigue which would inflate the association between those variables.

#### Patient-reported psychosocial data

We hypothesized based on previously published studies in SLE [23] and SSc [5] that coping skills and social support are possible determinants of fatigue severity. Illness behavior and social support were recorded by standard psychometric instruments. Coping with disease was evaluated with the Illness Behavior Questionnaire (IBQ) [24]. IBQ is a 62-item instrument with a summary score ranging from zero to 35. Higher scores indicate less appropriate illness behaviors. Social support was assessed by the Interpersonal Support Evaluation List (ISEL), a 40-item validated instrument with summary score of zero to ten [25]. Higher scores indicated better social support.

In confirmation of our previous findings [17], Fatigue Severity Scale and all psychometric instruments demonstrated adequate internal consistency reliability in the GENISOS cohort. FSS showed an adequate internal consistency as demonstrated by a Cronbach's Alpha value of 0.9. Social support measured by the ISEL questionnaire had a Cronbach's Alpha of 0.87. IBQ showed Cronbach's Alphas of 0.85.

### Statistical analysis

The investigated outcome was the sequentially obtained FSS scores. We utilized generalized linear mixed models (GLMMS) for all our analyses to evaluate the effects of the measured baseline variables on sequentially obtained FSS scores. We treated patients as a sample from a larger population and modeled between patient variability in FSS as a random intercept. We also modeled between patient variability in the change of FSS over time by a random slope (i.e., we estimated a separate slope for each patient). We accounted for the correlations among random effect parameters by an independent covariance matrix. Exchangeable or unstructured covariance matrices did not improve model fit evaluated by the Bayesian Information Criterion (BIC). Generally, mixed-effect models allow inclusion of all data points in the analysis and can also be used when some data points are missing.

We first investigated the relationship of baseline demographic, clinical, and psychosocial variables to sequentially obtained FSS in the univariable model. Subsequently, we built a multivariable model of objective clinical data. In general, this multivariable model is less susceptible to problems arising from a bidirectional relationship between the independent variables and the FSS than models that include patient-reported independent variables. We first included all objective clinical variables showing a univariable association with p<0.1 in the multivariable model. Then, the number of variables was reduced utilizing a forward hierarchical variable selection strategy. This variable selection approach was chosen to decrease the effect of multi-colinearity in our analysis.

We next conducted a hierarchical modeling with successive conceptual blocks to evaluate whether demographic, clinical and psychosocial variables independently contribute to FSS. The independent variables with a p<0.1 in the univariable analysis were added into the analysis in the following successive conceptual blocks: demographic variables, objective clinical manifestations, self-reported clinical outcomes (pain), and psychosocial variables (Figure S1). The model fit was assessed after addition of each conceptual block by using Bayesian Information Criterion (BIC). Lower BIC values indicate better model fit. BIC values are interpreted as very strong evidence for better fit if the new value is >10 lower than the previous value. This approach tested the proposition that each conceptual block independently predicts the sequentially obtained FSS scores and is not merely a mediator of the previous variable blocks.

The final multivariable model was built following the above described forward hierarchical variable selection strategy after inclusion of relevant demographic, clinical and psychosocial variables.

We also investigated the predictors of rate of change in FSS over time. For this purpose, the interaction term of the independent variable with the time-in-study was investigated. A baseline variable considered a predictor of change in FSS over time if the interaction term between the variable and the time-in-study was significant. The sign and magnitude of the interaction term coefficient show the direction and magnitude of the change over time.

All the statistical analyses were performed with STATA 11 (StataCorp, College Station, TX). The hypothesis testing was 2-sided with a p≤0.05 significance level.

## Results

### Sample characteristics

Between January 1998 and October 2009, 266 patients were enrolled in the GENISOS cohort. The mean (SD) follow-up time was 3.8 (3.4) years, ranging up to 11.4 years. The FSS measurement was not available in 10 patients. In this study, 1090 FSS scores belonging to 256 patients were analyzed. A total of 213 patients had at least one follow up FSS measurement. Out of remaining 43 patients, 6 were recent enrollees, 15 died, and 22 were lost to follow up.

The mean age (SD) of patients was 48.6 (13.3) years at enrollment, 83% were female. The proportions of Caucasian, African American and Hispanic patients were 47%, 20% and 29%, respectively. About 41% had limited cutaneous involvement. The mean disease duration (SD) at enrollment was 2.5 (1.6) years. Table 1 presents the baseline demographic, clinical, psychosocial characteristics of GENISOS cohort. Further details have been published previously.

**Table 1:** Population characteristics at baseline study visit.

Age, mean (SD), years	48.6(13.3)
Gender, female, *n* (%)	221 (83.1)
Ethnicity, *n* (%)	
*Caucasian*	125 (46.9)
*Hispanic*	77 (28.9)
*African-American*	54 (20.3)
*Other*	10 (3.9)
Disease duration, mean (SD), years	2.5 (1.6)
Cutaneous involvement, diffuse, *n* (%)	156 (58.6)
Autoantibody profile, *n* (%)	
*Anti-centromere antibody*	32 (12.0)
*Anti-topoisomerase antibody*	49 (18.4)
*Anti-polymerase III antibody*	62 (23.3)
*Anti-ribonucleic protein antibody*	30 (11.3)
Modified Rodnan Skin Score (MRSS), mean (SD)	15.8 (11.8)
Fatigue Severity Scale (FSS) score, mean (SD)	4.7 (0.9)
Interpersonal Support Evaluation List (ISEL) score, mean (SD)	8.1 (1.6)

### Progression of fatigue over time

**Figure 1:**
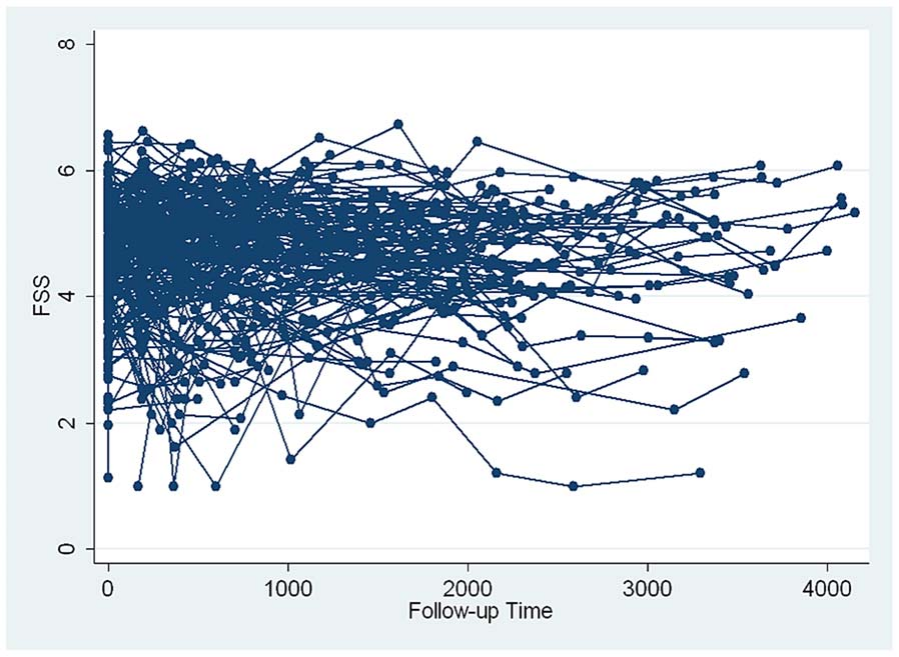
Course of Fatigue Severity Scale (FSS) scores in individual patients followed in the GENISOS cohort. X axis: follow-up time in days; Y axis: FSS scores.

**Figure 2:**
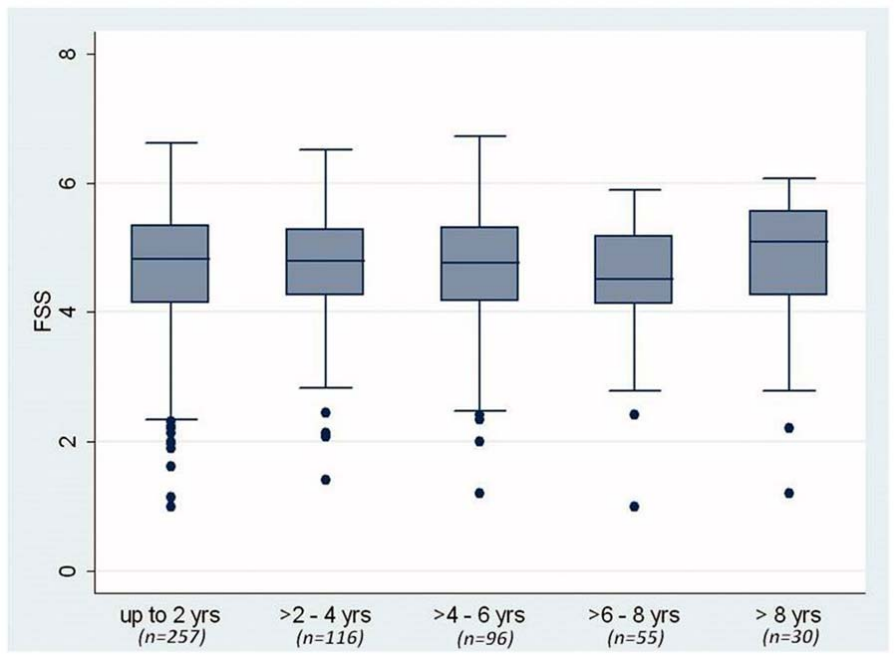
Course of Fatigue Severity Scale (FSS) scores over 2 year intervals of follow up time. Data are presented in box plots. Each box represents the 25th to 75th percentile. The length of the box is the interquartile range (IQR). The line inside the box represents the median. Whiskers reprsent 1.5 times the upper and lower IQRs. Circles indicate individual outliers. N is the number of patients who had at least one FSS measurement during the time interval.

At enrollment, the mean FSS (SD) score was 4.7 (0.9), ranging from 1.1 to 6.6. To determine if FSS scores change over time in SSc patients, sequentially obtained FSS scores from each individual in the GENISOS cohort were plotted over time in Figure 1. This demonstrated that the FSS score fluctuated in some individuals over time but the FSS levels did not show a consistent trend of change during the follow up time in the overall cohort. This was verified by the fact that time-in-study was not associated with a decline or increase in the sequentially obtained FSS levels (p = 0.221). Figure 2 graphically illustrates that the FSS scores did not change in 2-year intervals of follow-up time. Furthermore, our data did not indicate that mortality has influenced the observed course of fatigue because the vital status (dead versus alive) was neither predictive of differential levels of serially measured FSS (p = 0.761), nor it was a predictor of change in FSS (p = 0.992).

Based on the first and last available FSS measurement, 108 patients (50.7%) showed improved fatigue severity while 103 (48.4%) experienced worsening of fatigue severity. Two patients (0.9%) had the same FSS level on the last follow up visit. A minimally clinically important difference (MCID) for FSS has not been defined for patients with SSc. Therefore, we cannot report what percentage of patients had a clinically important change in FSS over time.

### Univariable predictors of sequentially obtained FSS

Among demographic variables, only current exercise was a significant predictor of sequentially obtained FSS scores (negative relationship) while gender, age, current smoking and ethnicity did not show a significant relationship to the outcome variable.

The presence of the following baseline clinical variables was a significant predictor of longitudinal FSS measurements: Diarrhea, dysphagia, anti-U1 RNP antibody, small joint contracture, higher serum creatinine level, and higher Medsger Gastrointestinal Index were associated with higher sequentially obtained FSS scores (increasing fatigue severity).

Both patient-reported clinical outcomes (VAS pain and dyspnea) were also associated with higher FSS. Among baseline psychosocial measures, maladaptive behavior (higher IBQ) was a significant predictor of higher longitudinal FSS scores. Detailed results of univariable analyses are shown in Table 2 and Table S1.

**Table 2:** Univariable analysis of demographic, clinical manifestation, patient-reported clinical outcomes, and psychosocial variables.

	*Main effect*		*Interaction between independent variable and time-in-study*	
Independent Variable	*b* (95% CI)	*p*-value	*b* (95% CI)	*p*-value
Follow-up time	−0.01 (−0.04, 0.01)	0.221		
**Demographic**				
Age	0.01 (−0.01, 0.01)	0.793	0.01 (−0.01,0.01)	0.31
Gender, female	0.08 (−0.20, 0.35)	0.579	−0.03(−0.09, 0.02)	0.255
Ethnicity, Caucasian	0.17 (−0.04, 0.37)	0.111	0.02(−0.02, 0.07)	0.287
Exercise habits	−0.31 (−0.52, −0.09)	0.004	0.02 (−0.02, 0.07)	0.367
Marital Status	−0.18 (−0.38−0.03)	0.093	0.01 (−0.04, 0.05)	0.919
**Clinical manifestations**				
Disease duration	0.01 (−0.05, 0.08)	0.658	0.01 (−0.01, 0.02)	0.752
Diffuse cutaneous involvement	−0.20 (−0.41, 0.01)	0.059	−0.01 (−0.05, 0.04)	0.971
Dysphagia	0.27 (0.07, 0.47)	0.009	−0.03 (−0.07, 0.02)	0.232
Diarrhea	0.28 (0.08, 0.48)	0.006	−0.03 (−0.07, 0.02)	0.221
BMI*	0.01 (−0.01, 0.02)	0.554	0.01 (−0.01, 0.01)	0.401
Small joint contracture	0.32 (0.05, 0.59)	0.021	−0.02 (−0.08, 0.04)	0.587
mRSS**	0.01 (−0.01, 0.02)	0.251	−0.01 (−0.01, 0.01)	0.947
No of comorbidities	0.06 (−0.01, 0.12)	0.089	0.01 (−0.01, 0.02)	0.954
Serum creatinine level	0.19 (0.02, 0.36)	0.033	−0.01 (−0.07, 0.06)	0.928
Hematocrit	0.01 (−0.02, 0.03)	0.889	0.01 (−0.01, 0.01)	0.106
Cardiac involvement	0.31 (−0.01, 0.62)	0.051	−0.02 (−0.1, 0.05)	0.538
Antibody profile				
*Anti-centromere antibody*	−0.16 (−0.47, 0.16)	0.329	0.01(−0.06, 0.08)	0.782
*Anti-topoisomerase antibody*	−0.12 (−0.39, 0.14)	0.359	0.03 (−0.03, 0.09)	0.298
*Anti-polymerase III antibody*	0.04 (−0.20, 0.27)	0.774	0.01 (−0.05, 0.05)	0.948
*U1-RNP*	0.42 (0.09, 0.74)	0.012	−0.04 (−0.12, 0.04)	0.376
FVC† % predicted value	−0.01 (−0.01, 0.01)	0.204	−0.01 (−0.01, 0.01)	0.06
DLco‡ % predicted value	−0.01 (−0.01, 0.01)	0.135	−0.01 (−0.01, 0)	0.013
Medsger Severity Index				
*General*	0.02 (−0.11, 0.15)	0.721	0.01 (−0.03, 0.03)	0.969
*Perivascular*	0.01 (−0.09, 0.10)	0.956	−0.01 (−0.03, 0.12)	0.447
*Skin*	0.07 (−0.05, 0.19)	0.259	0.01 (−0.03, 0.03)	0.992
*Joint*	0.08 (−0.01, 0.16)	0.059	0.01 (−0.02, 0.02)	0.915
*Muscle*	0.19 (−0.06, 0.43)	0.133	0.01 (−0.04, 0.06)	0.713
*GI Tract*	0.23 (0.08, 0.39)	0.004	0.01 (−0.4, 0.05)	0.819
*Lung*	0.04 (−0.05, 0.14)	0.346	0.01 (−0.01, 0.03)	0.250
*Heart*	0.06 (−0.09, 0.19)	0.439	0.03 (−0.01, 0.07)	0.208
*Kidney*	0.20 (−0.03, 0.44)	0.090	0.06 (−0.03, 0.15)	0.21
**Patient-reported clinical outcome**				
VASβ for pain	0.06 (0.03, 0.09)	<0.001	−0.01 (−0.01, 0.01)	0.522
VASβ for dypnea	0.09 (0.05, 0.12)	<0.001	−0.01 (−0.01, 0.01)	0.994
**Psychosocial measures**				
IBQΩ	0.06 (0.04, 0.08)	<0.001	−0.01 (−0.01, 0.01)	0.093
ISELΨ	−0.01 (−0.08, 0.06)	0.787	−0.01 (−0.02, 0.02)	0.959

*BMI: Body mass index;

**mRSS: modified Rodnan Skin Score;

† FVC: Forced vital capacity;

‡ DLco: Diffuse capacity of the lung for carbon monoxide;

β VAS: Visual Analogue Scale;

Ω IBQ: Illness Behavior Questionnaire;

Ψ ISEL: Interpersonal Support Evaluation List.

### Independent clinical predictors of sequentially obtained FSS

We next identified independent objective clinical correlates of sequentially obtained FSS, utilizing a forward hierarchical variable selection. In this multivariable analysis, higher Medsger Gastrointestinal (p = 0.006) and Joint (p = 0.024) Severity Indices and presence of anti-U1 RNP antibodies (p = 0.024) were independent predictors of higher FSS (Table 3).

**Table 3:** Multivariable analysis of objective clinical predictors of sequentially obtained FSS.

	Regression coefficient (95% CI)	*p*-value
Medsger Severity Index - GI tract	0.22 (0.06, 0.38)	0.006
Medsger Severity Index - Joint	0.09 (0.01, 0.17)	0.024
U1-RNP*	0.37 (0.05, 0.7)	0.024

*U1-RNP: Anti-U1 ribonucleoprotein antibodies.

### Successive conceptual blocks predicting sequentially obtained FSS

We next examined the predictive significance of baseline characteristics grouped in the following conceptual blocks: 1) demographic; 2) objective clinical manifestations; 3) patient-reported clinical outcomes; 4) psychosocial variables. In the following models, only the baseline variables were included that predicted the longitudinal FSS with p-values<0.1 in the univariable analysis. The results of this successive conceptual block modeling are shown in Figure S1 and Table S2.

In model 1, the relevant demographic variables were examined. This model had a BIC of 2381 (p = 0.007). In model 2, the relevant clinical variables were added to the previous model which resulted in an improved BIC of 2181 (delta = 200, p<0.001). This indicated that the addition of objective clinical variables led to a substantially better model fit. In model 3, we added the patient-reported clinical outcome, VAS for pain to the previous two blocks. This model also resulted in a better model fit as indicated by a BIC of 2132 (delta = 49, p<0.001). The VAS dyspnea was not included in this model because of concerns regarding a strong bidirectional relationship between perceived dyspnea and fatigue severity. In the last model, the relevant patient-reported psychosocial data were added to the previous conceptual blocks. Model 4 had the lowest BIC (2117) and showed a strong evidence for better model fit compared to Model 3 (delta = 15, p<0.001).

This blockwise hierarchical modeling strategy indicated that each successive block (demographic, objective clinical, patient-reported clinical, and psychosocial variables) had independent predictive significance for sequentially obtained FSS and was not merely a mediator of previous blocks.

### Independent predictors of sequentially obtained FSS (final model)

All relevant demographic, clinical, and psychosocial variables were included in the final model (Table 4). Following a forward variable selection strategy, VAS for pain (p = 0.006), maladaptive coping skills captured by higher IBQ score (p<0.001), and Medsger Gastrointestinal Severity Index (p = 0.009) were independent predictors of higher sequentially obtained FSS scores.

**Table 4:** Multivariable analysis of independent demographics, clinical, and patient-reported clinical outcome, and psychosocial predictors of longitudinally obtained FSS scores.

	Regression coefficient (95% CI)	*p*-value
VAS* for pain	0.04 (0.01, 0.07)	0.006
IBQ**	0.05 (0.03, 0.07)	<0.001
Medsger Severity Index - GI tract	0.2 (0.05, 0.35)	0.009

*VAS: Visual Analogue Scale.

**IBQ: Illness behavior questionnaire.

### Predictor of rate of change in FSS over time

In the last step, we investigated the predictive significance of baseline variables for rate of change in FSS over time. As shown in Table 2 and Table S1, patient's baseline DLco% predicted level was the only significant predictor of change in FSS over time (p = 0.013). Patients with higher DLco% predicted levels had a decline in FSS whereas patients with lower DLco% predicted levels experienced an increase in FSS over time. Similar trends were observed for baseline FVC% predicted (p = 0.06).

## Discussion

In this study, we examined the course of fatigue severity in a large, multi-ethnic cohort of early SSc patients. To our knowledge, the current study represents the first longitudinal examination of fatigue in SSc. FSS levels did not increase or decrease during the follow up time in the overall cohort, though patients with lower baseline DLco levels experienced an increase in their fatigue severity over time. Demographic, clinical, and psychosocial variables were all independent predictors of sequentially obtained FSS scores. Severity of GI and joint involvement and presence of anti-U1 RNP antibodies were independent predictors of FSS in the multivariable model of clinical factors. Baseline perceived pain levels, coping skills (IBQ), and GI involvement were independent predictors of longitudinal FSS in the final extended multivariable model.

Higher baseline scores of the Medsger Gastrointestinal Severity Index were predictive of higher FSS scores. This supports a reported association of GI involvement and higher fatigue scores in a cross sectional study of SSc patients [4]. GI involvement is very common in SSc patients and its strong association with depression has been previously reported [26], [27]. The association of GI dysmotility with fatigue severity may have several direct and indirect causes. Patients with diarrhea and decreased intestinal absorption might develop nutritional deficiencies with subsequent muscular and electrolyte abnormalities. Moreover, diarrhea and abdominal pain might interfere with sleep, resulting in higher fatigue scores. In patients with chronic fatigue syndrome, abdominal pain was stressful, but nocturnal diarrhea was found to further disrupt an already disrupted sleep pattern [28]. Fatigue is also a prominent feature of autoimmune diseases with primary GI manifestation such as Crohn's disease. In a randomized controlled study examining the effects of adalimumab therapy in patients with moderate to severe Crohn's disease, adalimumab maintenance therapy provided sustained improvement in fatigue severity compared to conventional immunosuppressive therapy [29].

In the current study, higher Medsger Joint Severity Index predicted higher FSS scores. The role of joint involvement as contributor to fatigue severity in SSc has not been previously reported. However, fatigue is also a prominent feature of other autoimmune diseases that primarily affect joints such as rheumatoid arthritis (RA) [30]. The effect of conventional disease modifying antirheumatic drugs (DMARD) on fatigue severity compared to placebo in RA has not been investigated but a significant improvement in fatigue severity in patients with moderate to severe RA was reported with adalimumab treatment compared to conventional DMARD therapy [31]. Furthermore, aerobic exercise, with most regimens consisting of 3 times weekly for 30–60 minutes exercises, was effective in treatment of fatigue in patients with RA (reviewed in [32]). Similar to RA, exercise habits were the only demographic variable predictive of fatigue severity in our study. This finding supports future interventional studies examining the efficacy of exercise regimens for treatment of fatigue in SSc.

Presence of U1-RNP antibodies were predictive of higher sequentially obtained FSS levels. Autoantibodies are important predictors of various disease manifestations in SSc [33], [34]. The association of SSc-related antibodies with fatigue severity has not been examined in previous publications. The U1-RNP antibodies are associated with overlap cases of SSc with other connective tissue diseases such as SLE and polymyositis. It is possible that experiencing features of multiple connective tissue diseases can lead to more severe fatigue.

In the final model, two patient-reported outcomes (pain and IBQ) were predictive of higher FSS levels. The blockwise hierarchical analysis indicated that patient-reported variables contributed to fatigue beyond the effect of clinical and demographic factors. Although the relationship of the patient-reported variables to FSS might be bidirectional (e.g. pain and IBQ influence FSS and vice versa). The reported multivariable model with objective clinical variables is least susceptible to problems arising from the bidirectional relationship between the predictor and outcome variables. However, we did not confine our study to objective clinical predictors because this would have ignored important subjective determinants of FSS. Furthermore, we did not only investigate the relationship of the above mentioned independent variables with the concomitantly obtained FSS levels but we also investigated whether they have predictive significance for FFS levels obtained on subsequent visits.

Inappropriate illness behavior (coping) captured by a higher IBQ score was an independent predictor of longitudinal FSS. The IBQ assesses a spectrum of illness behaviors or modes of perceiving, evaluating, or acting in relation to one's own state of health that may be in contradistinction to an accurate appraisal of the condition and prescribed treatment [24]. Similar to our results, the LUMINA study has demonstrated the association of higher IBQ scores with higher scores of perceived fatigue in SLE [23]. Furthermore, it has also been shown that higher IBQ scores reflecting worse coping behavior can affect the quality of life in SLE patients [14] . In patients with RA, group cognitive behavioral therapy for fatigue self-management (coping) was found be effective in treating fatigue severity in a recently published randomized controlled trial [35]. Our study provides further support for similar interventional studies in SSc, examining the efficacy of self management and coping strategies for treatment of fatigue.

Pain was another patient-reported variable that predicted higher FSS levels in our study. This finding is in agreement with longitudinal studies of fatigue in patient with SLE [23]. Pain in SSc can be caused by various disease manifestations such as joint pain, digital ulcer, heartburn, and tendon friction rub [36]. Better treatment of pain and more effective management of its underlying causes might alleviate fatigue severity in patient with SSc.

FSS scores did not increase or decrease during the follow up time in the overall cohort. Factors leading to worsening fatigue such as increasing age and disease damage might be counterbalanced by improving adaptive behaviors leading to stable longitudinal fatigue severity in SSc. A study of a longitudinal cohort of 122 patients with RA also reported that FSS scores did not change appreciably over time [37]. Furthermore, studies in patients with chronic fatigue syndrome indicated that patients with longer disease duration had better adaptive coping strategies than those with shorter disease duration, supporting the hypothesis that patients with chronic illnesses develop better coping skills for dealing with fatigue over time. Another possible explanation for stable longitudinal fatigue levels is that fatigue might be related to inherent perceived health or coping mechanisms. Although the success of exercise regimens [32], behavioral [35] and pharmacological [29], [31] interventions for treatment of fatigue in other rheumatic diseases indicates that this disease manifestation is modifiable and not solely related to related inherent and non-modifiable patient characteristics.

DLco% predicted was the only baseline variable that was predictive of change in fatigue severity. A similar trend was observed for FVC although it did not reach statistical significance. This finding indicates that patients with more extensive lung involvement are more likely to experience an increase in their fatigue levels over time. Several medications are effective in treatment of pulmonary arterial hypertension (reviewed in [38] ) and cyclophosphamide is beneficial for treatment of interstitial lung disease in SSc [39]. It is unclear whether treatment with these agents can lead to a reduction in fatigue severity in SSc. Furthermore, the role of pulmonary rehabilitation in treatment of lung impairment and fatigue also has not been investigated in SSc. In patients with chronic obstructive pulmonary disease, pulmonary rehabilitation for 3 months was effective for treatment of dyspnea and fatigue [40].

The current study had some limitations. The majority of study subjects were recruited from tertiary medical centers, which might skew the study population toward patients with more severe involvement. Furthermore, we did not have information on sleep disturbances in the GENISOS cohort, a factor that might be an independent predictor of fatigue in SSc. Furthermore, we did not use a designated questionnaire for capturing depressive symptoms in the GENISOS.

Fatigue is a prominent and debilitating problem for a large number of SSc patients. Our results indicate that potentially modifiable clinical and psychological factors predict longitudinal fatigue severity. Measures to decrease physical burden of disease such as respiratory, GI and joint involvement, as well as interventions focusing on improving coping skills and pain could potentially improve fatigue severity in SSc.

## Supporting Information

Figure S1 **Model structure of the blockwise hierarchical analysis.**
(DOC)

Table S1 **Univariable analysis of demographic, clinical, patient-reported clinical, and psychosocial variables.** Abbreviations: BMI: Body mass index; MRSS: modified Radnon Skin Score; FVC: Forced vital capacity; DLco = Diffuse capacity of the lung for carbon monoxide; VAS: visual analogue scale; IBQ: Illness Behavior Questionnaire; ISEL: Interpersonal Support Evaluation List.
(DOC)

Table S2 **Blockwise modeling of demographic, clinical, patient-reported clinical, and psychosocial predictors of longitudinal FSS (** ***variables with p<0.1 included*** **).** ^*^BIC: Bayesian Information Criterion.
(DOC)
