# Supplementary figures and images for: Predictors of Fatigue Severity in Early Systemic Sclerosis: A Prospective Longitudinal Study of the GENISOS Cohort
Source: PLoS One. 2011 Oct 14;6(10):e26061. doi: 10.1371/journal.pone.0026061 (PMC3193535; doi:10.1371/journal.pone.0026061)

Figure S1: Model structure of the blockwise hierarchical analysis


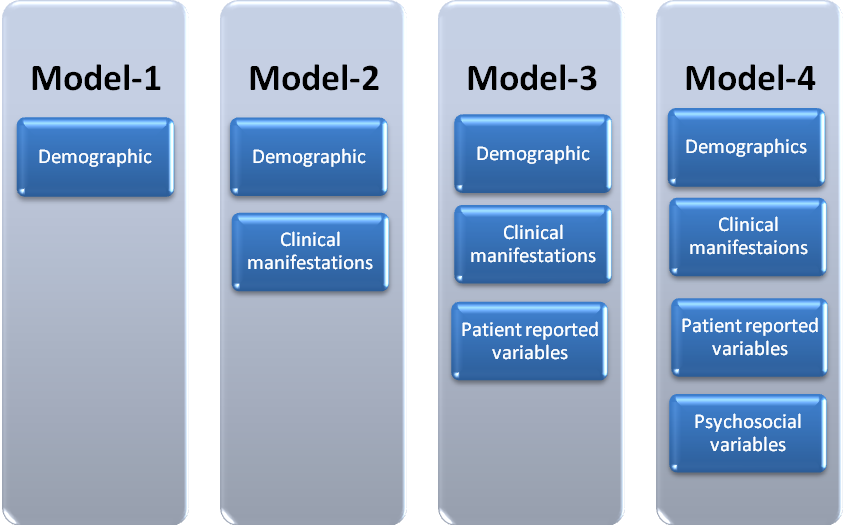

Supplement: Figure S1 — Model structure of the blockwise hierarchical analysis. (DOC) [file pone.0026061.s001.doc]
