# Supplementary material for: Predictors of Fatigue Severity in Early Systemic Sclerosis: A Prospective Longitudinal Study of the GENISOS Cohort
Source: PLoS One. 2011 Oct 14;6(10):e26061. doi: 10.1371/journal.pone.0026061 (PMC3193535; doi:10.1371/journal.pone.0026061)
Supplement: Table S1 — Univariable analysis of demographic, clinical, patient-reported clinical, and psychosocial variables. Abbreviations: BMI: Body mass index; MRSS: modified Radnon Skin Score; FVC: Forced vital capacity; DLco = Diffuse capacity of the lung for carbon monoxide; VAS: visual analogue scale; IBQ: Illness Behavior Questionnaire; ISEL: Interpersonal Support Evaluation List. (DOC) [file pone.0026061.s002.doc]

| **Table S1 -** Univariate analysis of demographic, clinical, patient-reported clinical, and psychosocial variables | | | | |
| --- | --- | --- | --- | --- |
|  | Main effect | | *Interaction between independent variable and time in Study* | |
| Independent Variable | *b* (95% CI) | *P* value | *b* (95% CI) | *P value* |
| Elapse year | -0.01 (-0.04, 0.01) | 0.221 |  |  |
| **Demographic** |  |  |  |  |
| Age | 0.01 (-0.01, 0.01) | 0.793 | 0.01 (-0.01,0.01) | 0.31 |
| Gender, female | 0.08 (-0.20, 0.35) | 0.579 | -0.03(-0.09, 0.02) | 0.255 |
| Ethnicity, Caucasian | 0.17 (-0.04, 0.37) | 0.111 | 0.02(-0.02, 0.07) | 0.287 |
| Education level | 0.07 (-0.15, 0.28) | 0.546 | 0.01(-0.04, 0.05) | 0.807 |
| Current smoking | 0.15 (-0.12, 0.41) | 0.277 |  |  |
| Marital status, being married or in marriage-like relationship | -0.18 (-0.39, 0.03) | 0.093 | 0.01 (-0.04, 0.05) | 0.919 |
| Exercise habits | - 0.31 (-0.52, -0.09) | 0.004 | 0.02 (-0.02, 0.07) | 0.367 |
| **Clinical manifestations** |  |  |  |  |
| Disease duration (since 1^st^ non-Raynaud’s) | 0.01 (-0.05, 0.08) | 0.658 | 0.01 (-0.01, 0.02) | 0.752 |
| Disease duration (since 1^st^ symptom attributable to SSc) | -0.01 (-0.02, 0.01) | 0.701 | -0.01 (-0.01, 0.01) | 0.989 |
| Diffuse cutaneous involvement | -0.20 (-0.41, 0.01) | 0.059 | -0.01 (-0.05, 0.04) | 0.971 |
| Dysphagia | 0.27 (0.07, 0.47) | 0.009 | -0.03 (-0.07, 0.02) | 0.232 |
| Diarrhea | 0.28 (0.08, 0.48) | 0.006 | -0.03 (-0.07, 0.02) | 0.221 |
| Cardiac involvement | 0.31 (-0.01, 0.62) | 0.051 | -0.02 (-0.1, 0.05) | 0.538 |
| BMI | 0.01 (-0.01, 0.02) | 0.554 | 0.01 (-0.01, 0.01) | 0.401 |
| Small joint contracture | 0.32 (0.05, 0.59) | 0.021 | -0.02 (-0.08, 0.04) | 0.587 |
| mRSS | 0.01 (-0.01, 0.02) | 0.251 | -0.01 (-0.01, 0.01) | 0.947 |
| No of comorbidities | 0.06 (-0.01, 0.12) | 0.089 | 0.01 (-0.01, 0.02) | 0.954 |
| Hematocrit | 0.01 (-0.02, 0.03) | 0.889 | 0.01 (-0.01, 0.01) | 0.106 |
| Myositis | 0.07(-0.28, 0.41) | 0.707 | -0.01 (-0.08, 0.05) | 0.66 |
| Serum creatinine level | 0.19 (0.02, 0.36) | 0.033 | -0.01 (-0.07, 0.06) | 0.928 |
| Antibody profile |  |  |  |  |
| *Anti-centromere antibody* | -0.16 (-0.47, 0.16) | 0.329 | 0.01(-0.06, 0.08) | 0.782 |
| *Anti-topoisomerase* | -0.12 (-0.39, 0.14) | 0.359 | 0.03 (-0.03, 0.09) | 0.298 |
| *Anti-polymerase III* | 0.04 (-0.20, 0.27) | 0.774 | 0.01 (-0.05, 0.05) | 0.948 |
| *Ro (SS-A)* | -0.15 (-0.70, 0.41) | 0.605 | 0.09 (-0.03, 0.21) | 0.138 |
| *Anti-RNP* | 0.42 (0.09, 0.74) | 0.012 | -0.04 (-0.12, 0.04) | 0.376 |
| FVC % predicted value | -0.01 (-0.01, 0.01) | 0.204 | -0.01 (-0.01, 0.01) | 0.06 |
| DLco % predicted value | -0.01 (-0.01, 0.01) | 0.135 | -0.01 (-0.01,-0.01) | 0.013 |
| Medsger Severity Index |  |  |  |  |
| *General* | 0.02 (-0.11, 0.15) | 0.721 | 0.01 (-0.03, 0.03) | 0.969 |
| *Perivascular* | 0.01 (-0.09, 0.10) | 0.956 | -0.01 (-0.03, 0.12) | 0.447 |
| *Skin* | 0.07 (-0.05, 0.19) | 0.259 | 0.01 (-0.03, 0.03) | 0.992 |
| *Joint* | 0.08 (-0.01, 0.16) | 0.059 | 0.01 (-0.02, 0.02) | 0.915 |
| *Muscle* | 0.19 (-0.06, 0.43) | 0.133 | 0.01 (-0.04, 0.06) | 0.713 |
| *GI Tract* | 0.23 (0.08, 0.39) | 0.004 | 0.01 (-0.4, 0.05) | 0.819 |
| *Lung* | 0.04 (-0.05, 0.14) | 0.346 | 0.01 (-0.01, 0.03) | 0.250 |
| *Heart* | 0.06 (-0.09, 0.19) | 0.439 | 0.03 (-0.01, 0.07) | 0.208 |
| *Kidney* | 0.20 (-0.03, 0.44) | 0.090 | 0.06 (-0.03, 0.15) | 0.21 |
| **Patient-reported clinical outcome** |  |  |  |  |
| VAS pain | 0.06 (0.03, 0.09) | <0.001 | -0.01 (-0.01, 0.01) | 0.522 |
| VAS dyspnea | 0.09 (0.05, 0.12) | <0.001 | -0.01 (-0.01, 0.01) | 0.994 |
| **Psychosocial measures** |  |  |  |  |
| IBQ score | 0.06 (0.04, 0.08) | <0.001 | -0.01 (-0.01, 0.01) | 0.093 |
| ISEL score | -0.01 (-0.08, 0.06) | 0.787 | -0.01 (-0.02, 0.02) | 0.959 |
| Abbreviations: BMI: Body mass index; MRSS: modified Radnon Skin Score; FVC: Forced vital capacity; DLco= Diffuse capacity of the lung for carbon monoxide; VAS: visual analogue scale; IBQ: Illness Behavior Questionnaire; ISEL: Interpersonal Support Evaluation List | | | | |
