# Supplementary material for: Predictors of Fatigue Severity in Early Systemic Sclerosis: A Prospective Longitudinal Study of the GENISOS Cohort
Source: PLoS One. 2011 Oct 14;6(10):e26061. doi: 10.1371/journal.pone.0026061 (PMC3193535; doi:10.1371/journal.pone.0026061)
Supplement: Table S2 — Blockwise modeling of demographic, clinical, patient-reported clinical, and psychosocial predictors of longitudinal FSS ( variables with p<0.1 included ). *BIC: Bayesian Information Criterion. (DOC) [file pone.0026061.s003.doc]

|  | **Model 1**  **Demographic** | **Model 2**  **Demographic and objective clinical manifestations** | **Model 3**  **Demographic, objective clinical, and patient-reported clinical outcomes** | **Model 4**  **Demographic, objective clinical, patient-reported clinical outcomes, and psychosocial factors** |
| --- | --- | --- | --- | --- |
| Marital Status | -0.17 (-0.38, 0.04) | -0.09 (-0.30, 0.13) | -0.05 (-0.27, 0.17) | -0.01 (-0.22, 0.21) |
| Exercise Habits | -0.30 (-0.51, 0.09) | -0.32 (-0.54, -0.10) | -0.3 (-0.52, -0.08) | -0.15 (-0.37, 0.07) |
| Diffuse Cutaneous Involvement |  | -0.14 (-0.36, 0.09) | 0.17 (-0.06, 0.4) | 0.06 (-0.16, 0.29) |
| Small Joint Contracture |  | 0.24 (-0.07, 0.54) | 0.17 (-0.14, 0.49) | 0.24 (-0.07, 0.55) |
| Diarrhea |  | 0.13 (-0.09, 0.34) | 0.11 (-0.11, 0.33) | 0.09 (-0.12, 0.3) |
| Cardiac Involvement |  | 0.11 (-0.22, 0.43) | 0.11 (-0.21, 0.44) | 0.15 (-0.16, 0.46) |
| RNP |  | 0.47 (0.11, 0.82) | 0.42 (0.06, 0.78) | 0.36 (0.01, 0.71) |
| Serum Creatinine |  | 0.12 (-0.08, 0.32) | 0.12 (-0.09, 0.32) | 0.08 (-0.11, 0.28) |
| Medsger Severity Index, GI tract |  | 0.14 (-0.03, 0.32) | 0.15 (-0.03, 0.33) | 0.14 (-0.03, 0.31) |
| Medsger Severity Index, Joint |  | 0.05 (-0.04, 0.14) | 0.05 (-0.04, 0.14) | 0.05 (-0.04, 0.14) |
| Medsger Severity Index, Kidney |  | -0.02 (-0.29, 0.25) | -0.03 (-0.31, 0.24) | -0.07 (-0.33, 0.19) |
| No. of Comorbidities |  | 0.02 (-0.05, 0.10) | 0.02 (-0.05, 0.1) | 0.03 (-0.05, 0.1) |
| VAS-Pain |  |  | 0.03 (-0.01, 0.06) | 0.01 (-0.02, 0.05) |
| IBQ-Score |  |  |  | 0.04 (0.03, 0.06) |
| *p-value* | **0.007** | **<0.001** | **<0.001** | **<0.001** |
| BIC^*^ | **2380.8** | **2180.5** | **2131.9** | **2116.7** |

Table S2- Blockwise modeling of demographic, clinical, patient-reported clinical, and psychosocial predictors of longitudinal FSS (*variables with p <0.1 included*)

^*^BIC: Bayesian Information Criterion
